# MiRAGDB: A Knowledgebase of RAG Regulators

**DOI:** 10.3389/fimmu.2022.863110

**Published:** 2022-03-24

**Authors:** Sagar Sanjiv Desai, Saurabh Whadgar, Sathees C. Raghavan, Bibha Choudhary

**Affiliations:** ^1^Department of Biotechnology and Bioinformatics, Institute of Bioinformatics and Applied Biotechnology, Bangalore, India; ^2^Graduate Student Registered Under Manipal Academy of Higher Education, Manipal, India; ^3^Department of Biochemistry, Indian Institute of Science, Bangalore, India

**Keywords:** RAG1, RAG2, miRNA, transcriptome, database, regulation, CLL, T-ALL

## Abstract

RAG1 and RAG2 genes generate diversity in immunoglobulin and TCR genes by initiating the process of V-D-J recombination. RAGs recognize specific sequences (heptamer-nonamer) to generate a diversity of immunoglobulins. RAG expression is limited to early B and T cell developmental stages. Aberrant expression of RAG can lead to double strand breaks and translocations as observed in leukemia and lymphoma. The expression of RAG is tightly regulated at the transcriptional and posttranscriptional levels. MicroRNAs (miRNAs) are small non-coding RNAs that are involved in the post-transcriptional regulation of gene expression. This study aimed to identify and catalog RAG regulation by miRNA during normal development and cancer. NGS data from normal B-cell and T-cell developmental stages and blood cancer samples have been analyzed for the expression of miRNAs against RAG1 (1,173 against human RAG1 and 749 against mouse RAG1). The analyzed data has been organized to retrieve the miRNA and mRNA expression of various RAG regulators (10 transcription factors and interacting partners) in normal and diseased states. The database allows users to navigate through the human and mouse RAG regulators, visualize and plot expression. miRAGDB is freely available and can be accessed at http://52.4.112.252/shiny/miragdb/.

## Introduction

Recombination activating gene 1 (RAG1), along with its partner RAG2, constitutes a lymphoid-specific protein complex involved in initiating V(D)J recombination, a DNA rearrangement-based process that assembles diverse immunoglobulins and T cell receptor genes (1–3). These antigen receptor genes are formed by the recombination of variable (V), diversity (D), and joining (J) gene segments in the early stages of developing B and T lymphocytes (1, 4–6). Each V, D, and J segment is flanked by specific sequences called recombination signal sequences (RSSs). Each RSS consists of mildly conserved heptamer and nonamer sequences, separated by a spacer region of 12 or 23 base pairs and RAG complex recognizes the sequence to create a nick. The recombination efficiency is at a maximum only when it is between the 12 and 23 RSSs (12/23 rule). RAGs cleave the DNA segments using the 12/23 rule in conjunction with the heptamer and nonamer sequences of the respective RSSs (1, 7, 8). The expression and normal physiological function of RAGs are limited to the early stages of B cell development followed by a decline in expression in mature stages (4, 9).

Additionally, in a pathological scenario, RAGs are known to play a central role in generating chromosomal translocations in lymphoid tissues. Inter-chromosomal translocations are a byproduct of V(D)J recombination when RAGs act in a non-physiological state *via* cleavage at cryptic RSSs and non-B DNA structures (7, 9–12). Various RAG cleavage mechanisms exist apart from the physiological function and cause single stranded or double stranded nicks (13). The nicks in DNA form double-stranded breaks, resulting in cell death or translocations leading to aberrant conditions, including leukemias and lymphomas (9, 14). Aberrant RAG expression promotes lymphocyte malignancies *via* chromosomal translocations (e.g., t (11,14), t (10,14), t (14,18)) (15, 16). RAG mediated translocations in humans and mice have been linked to pro-B cell lymphomas, GCB-derived lymphomas, Follicular lymphomas, mantle cell lymphomas, B cell Acute lymphoblastic leukemia (17, 18).

The stage-specific function of RAG1 and RAG2 in lymphoid progenitors and their well-studied role in genomic instability warrants a regulatory mechanism for their expression. At the transcript level, RAG1 and RAG2 expression is regulated by several transcription factors, such as FOXO1, PAX5, IKAROS, and E2A (19). Deletion of IKZF1 gene, which codes for IKAROS protein, has been linked to high expression of RAG1 in adult B-ALL, hypothesizing the role of aberrant RAG expression, in absence of IKAROS, as a driver of ALL, whereas RAG1 and RAG2 have been identified as downstream, activating targets of NOTCH1 in T-ALL (20, 21).

Another central regulatory mechanism is the involvement of microRNAs. MicroRNAs (miRNAs) are small RNA molecules which are 18-24 nucleotide long. They regulate gene expression by binding to 3’UTR regions of mRNA molecules and suppress their expression by degrading them or blocking their translation (22, 23). Several studies have linked the regulatory role of miRNAs in lymphoid tissues and their cancers (24). One of the first reports of miRNA mediated immune cell regulation talks about B cell lineage specificity of miR-181 in murine bone marrow (24, 25). The widely studied miR-17-92 cluster is known to induce T cell survival and differentiation by suppressing pro-apoptotic proteins (26). MiR-29 miRNA family is crucial to the survival and differentiation of lymphoid cells. A recent study has shown that miR-29c regulates RAG1 expression in a developmental stage specific manner and has an inverse correlation of its expression with that of RAG1 in T-ALL and CLL patients (27, 28). This is the story of just one miRNA with its effect on RAG1 and subsequently on the behavior of lymphoid cells, but it is a well-known fact that multiple miRNAs can regulate one common target gene (29, 30) and RAGs need not be an exception, even more so because RAG1 and RAG2 have a genome wide participation and multiple functional modalities.

Having established the fact that RAGs are expressed only in the early stages of B and T cell development and pose a genome-wide threat in the context of pathological translocations, it is essential to study and collate information on its regulation at the transcriptional and translational level, that is, expression levels of miRNA and transcription factors associated with RAG1 and RAG2. In order to do so, we have profiled RAG1, RAG2 and their regulators in normal B and T cells and leukemia into a unique, comprehensive RAG specific database, miRAGDB. Apart from the visual representation of existing expression data, the database also offers a utility to upload data and plot a heatmap. Additionally, there is informative support in the form of links to MGI,UCSC, browser, UniProtKb, Entrez, Ensembl, OMIM, HGNC, databases and Atlas of Genetics and Cytogenetics in Oncology and Haematology which contain information on various RAG1 phenotypes.

## Implementation

### Datasets Used

Publicly available Total RNA and small RNA Transcriptome Sequencing data from Human and mouse B and T cell developmental stages and T-ALL and CLL Leukemia data (**Table 1**).

**Table 1:** Publicly available Transcriptome Datasets from NCBI-sra used for data collection and analysis.

Dataset	Organism	Data used	Type of sequencing
SRP002412 (31, 32)	Mouse	Developmental stages of B cells	small-RNA
SRP002958 (31)	Human	Developmental stages of B cells	small-RNA
SRP000729 (32, 33)	Human	Developmental stages of B cells	small-RNA
SRP026197 (34)	Mouse	Developmental stages of T cell	Total RNA
ERP110615	Mouse	Development of B cell stages	Total RNA
GSE66186 (35)	Human	Chronic Lymphocytic Leukaemia	small-RNA and mRNA
GSE110637 (36)	Human	T cell Acute lymphoblastic Leukaemia	Total RNA
GSE89978 (36, 37)	Human	T cell Acute lymphoblastic Leukaemia	small-RNA

### Computation of Transcript Expression

Raw reads from the small RNA sequencing datasets were checked for their quality using the FastQC(https://www.bioinformatics.babraham.ac.uk/projects/fastqc/). Based on phred score quality cut-off, low quality reads were trimmed from both the ends keeping a minimum of 18 nucleotides from an initial read length of 26-36 nucleotides. Post trimming the reads were mapped to the mouse reference (mm10) and human reference (hg38) genome, respectively using the Bowtie2 (38)aligner. The resulting SAM files were converted to compressed BAM format using the SAMtools package (39). In order to quantify the expression, the read counts per miRNA transcript were obtained using the BEDtools suite (40). The read counts were normalized by using the RPM method, i.e., RPM = number of reads mapped per transcript/(total number of mapped reads/10^6^). The rpm values were extracted and analyzed for difference in expression. The miRNA known to regulate RAG1 and RAG2 were obtained from miRDB (41) and TargetScan (42). The miRNAs targeting RAGs were obtained based on a custom prediction available in the databases based on the presence of seed sequences that bind to the 3’UTR of RAG1 and RAG2. The shortlisted miRNA were then checked for their expression in the small-RNA datasets (**Figure S1A**).

For the T-ALL and CLL patients’ datasets, Normalized Read count files for miRNA transcripts and mRNA transcripts from 15 CLL patients were downloaded from the GEO datasets mentioned in **Table 1**. Raw read count files for miRNA and mRNA transcripts for 48 T-ALL patients were downloaded from the GEO database. RPM values for miRNA and RPKM values for mRNA were calculated for both the datasets using the following formulae, RPM = read count/(total number of reads mapped/10^6^) and RPKM = read count/(transcript length/1000) ∗(total number of reads mapped/10^6^). The normalized values for RAG1, its interacting partners and the miRNA known to regulate RAG1 were plotted in a heatmap using the pheatmap package in R.

### Framework

The database has been structured and constructed using the Shiny package from RStudio^©^ (https://shiny.rstudio.com/). We are providing an offline and online version of the database; the online version is deployed on the Rstudio server (https://www.shinyapps.io/) which works on static TSV/CSV files resulting from the analysis which contains expression data. At the same time, the offline version works on SQLite database. The data is fetched and rendered in a table or graphical format on the UI based on the user query.

### Working of Shiny R Application

The application is written in 3 separate R files, namely ui.R, server.R and global.R. The ui.R renders user interface, whereas server.R does processing of data as per user input and global.R reads all CSV files required for the database (**Figure S1B**). List of packages used to read in and visualize the data are mentioned in **Figure S1C**.

## Database Organization and Access

Implemented using Shiny-R, miRAGDB is a multi-utility display interface specific to the expression data of RAG1, RAG2 and its regulators in normal and aberrant B and T cell conditions. The small RNA and Transcriptome data belong to two organisms, mice and humans distributed over various interactive utilities, which are as follows.

### Home Page

**Figure 1:**
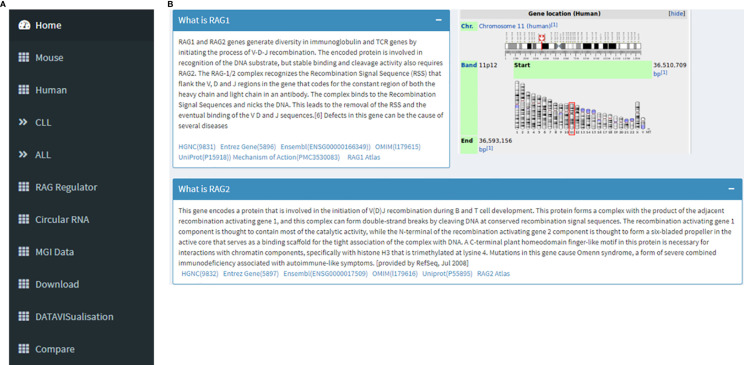
**(A)** Navigation panel that contains links to all the pages of the database. **(B)** This image collectively gives an introduction to RAG1 and RAG2, its function and its chromosomal location. It can also be seen that there are links to RAG1 related pages of HGNC, Entrez Gene, Ensembl, OMIM, UniProtKB, Atlas of Genetics and Cytogenetics in Oncology and Haematology.

The database URL lands on the “Home” page. On the left side there is a navigation panel that lets the user choose the next page they want (**Figure 1A**). This page displays information about RAG1, RAG2 and its genomic location on chr11, and its role in V(D)J recombination in B and T cells as part of the RAG nuclease complex. This page offers an option for the users to be able to navigate to the RAG1 related pages of HGNC, Entrez Gene, Ensembl, OMIM, UniProtKB and Atlas of Genetics and Cytogenetics in Oncology and Haematology databases along with a link to a diagram describing RAG mechanism through the “Mechanism of Action” tag (**Figure 1B**).

### Mouse Page

Based on a previous study from the lab, where the role of miRNA in the regulation of RAGs was investigated, small RNA-seq datasets consisting of mouse developmental stages of B and T cells were downloaded. Using a standard transcriptome analysis pipeline with mm10 as a reference genome for alignment, followed by calculation of normalized expression, miRNA regulating RAG1 and RAG2 were analyzed for the difference in expression across developmental stages of normal mouse B and T cells. It is known that expression of RAGs differs in a stage-dependent manner and hence, the miRNA expression data were analyzed for a corresponding inverse expression profile of miRNA. Transcriptome datasets from normal B and T cells were also obtained and analyzed (**Table 1**). These expression profiles are displayed on the mouse page of miRAGDB.

**Figure 2:**
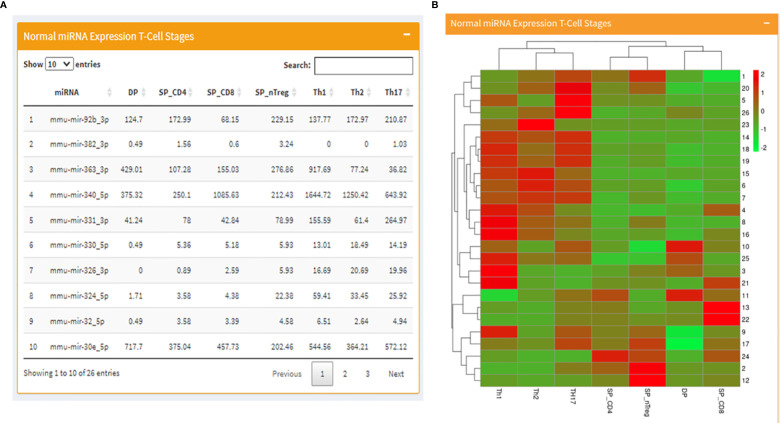
Mouse page of the database where **(A)** displays a table with RAG1 miRNA expression across T cell developmental stages and **(B)** contains the corresponding heatmap.

The page begins with a tabular display of RAG regulating miRNA expression values in normal T cell development stages on the left alongside a corresponding heatmap representing the same expression profiles on the right (**Figures 2A, B**). Following the miRNA expression, the page moves to tabulated RAG1 expression values in normal T-cell stages followed by a bar graph representing the same. In the end, just as it was displayed for T- cells, there is a side-by-side display of RAG1 and RAG2 regulating miRNA expression values in B cell development stages on the left and the corresponding heatmap on right.

### Human Page

**Figure 3:**
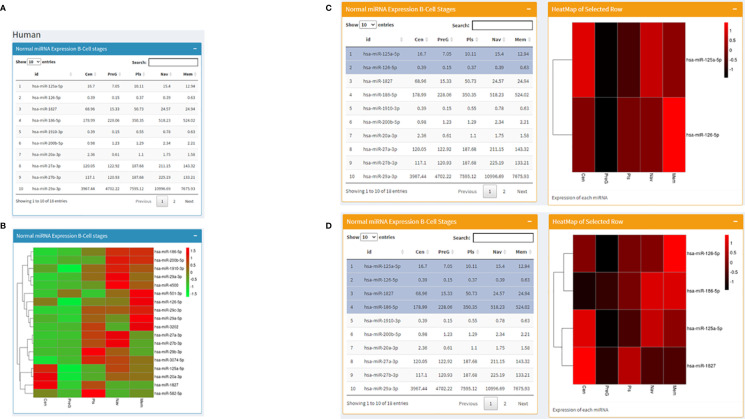
Human page of the database where **(A)** displays a table with RAG1 miRNA expression across normal B cell development stages and **(B)** contains the corresponding heatmap; **(C, D)** set of images represent the utility of the page where heatmaps corresponding to selected miRNA are displayed; **(C)** has 2 miRNAs selected and **(D)** has 4 miRNAs selected on the left along with the corresponding heatmaps on the right of the images.

Datasets consisting of small-RNA sequencing across human B cell development stages were downloaded (**Table 1**) and processed using hg19 as the human reference genome. Similar to the mouse page, this area displays miRNA expression profiles in tabular format along with a visualization in the heatmap and bar graph forms. The difference is that this page is rather interactive. In the beginning, it starts in the same way as the mouse page with miRNA expression values across stages of B cell development in a table on the left and a corresponding heatmap on the right (**Figures 3A, B**). Going forward, we present the same miRNA B cell data in the form of individual bar graphs available to the users as per their selection from a dropdown list of the miRNAs. Further, we present a fascinating utility where from a table of expression profiles of miRNA across B-cell development stages, the user can choose any number of miRNAs (at least two) in any order, and a corresponding heatmap representing the expression profile will be displayed on the right in that specific order (**Figures 3C, D**). However, the shade of the color corresponding to the expression value in the heatmap for every miRNA for each stage will differ slightly with the addition or removal of every miRNA. At the end just as in the mouse page, the human page displays expression levels of miRNA against RAG2 in Human B cell development stages.

### RAG Associated miRNA Expression in Aberrant B and T Cell Conditions

The following two pages deal with the expression of RAG1 and the miRNA regulating it in two datasets, Chronic lymphocytic leukemia, and T-cell Acute Lymphoblastic Leukemia, respectively.

**Figure 4:**
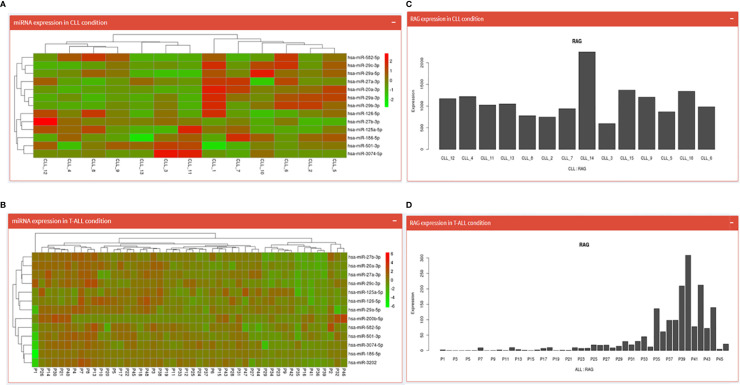
CLL and T-ALL pages; where **(A, B)** show the heatmaps of miRNA regulating RAG1 in CLL and T-ALL conditions while **(C, D)** depict normalized RAG1 expression values in these conditions.

Both the pages begin by displaying an expression table of RAG1 regulating miRNA across 15 CLL patient samples (17 miRNA spread across two pages) and 48 T-ALL samples. This table includes a search dialogue box where the user can search for one of the 17 miRNAs and obtain expression values specific to that miRNA. Further down is a heatmap of those miRNAs (**Figures 4A, B**) and at the end is a Bar graph showing RAG1 expression across the 15 CLL patients and 48 T-ALL patients (**Figures 4C, D**).

### Expression Profiles of RAG1, RAG2 and Its Interacting Partners

**Figure 5:**
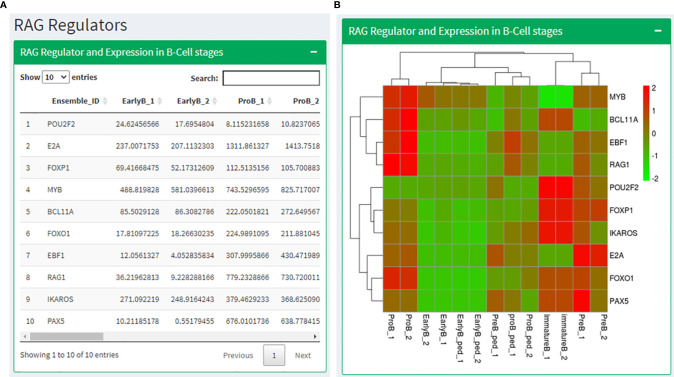
RAG regulators page where **(A)** shows a table with the expression values RAG1 interacting partners in B cell stages along with the corresponding heatmap in **(B)**.

Based on the literature study, we prepared a list of genes known to regulate and interact with RAG1 (POU2F2, E2A, FOXP1, MYB, BCL11A, FOXO1, EBF1, IKAROS, and PAX5) and RAG2 (BCL11A, E2A, PAX5, SATB1, CDK2, GATA3, IKAROS, RUNX1 and ETV6). We identified the expression profiles of these genes in human and mouse B cell development data and T cell development data. The RAG regulators page displays these expression values in a tabular format followed by a heatmap showing the same (**Figures 5A, B**). After that, the expression levels of genes known for RAG1 degradation and localization in B and T cell development data has been shown in a tabular as well as heatmap format. The next section shows expression data of RAG2 regulators in B and T cell development data across their development stages. The page ends with Expression of RAG regulators in CLL condition.

### Links to Additional Information About RAG1 and RAG2

**Figure 6:**
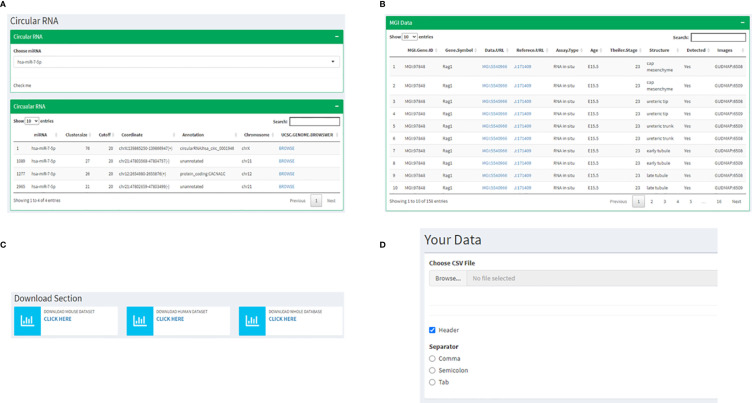
Instances of subsequent pages where **(A)** shows the utility of miRNA dropdown menu and a corresponding table listing the UCSC links to corresponding circRNA chromosomal locations; **(B)** represents the MGI page depicting a table with links to information related to RAG1 from the MGI database; **(C)** shows the page where all the data from the database can be downloaded and **(D)** represents the interactive utility where the users have an option to visualize their own data by uploading files.

The following two pages are curations of data from other resources with information specific to RAG1 and RAG2. Since a significant highlight of this database is miRNA involved in RAG1 and RAG2, on the next page, we have cataloged all the circular RNAs that are known to make a sponge with every miRNA that we have mentioned in the previous pages. This instance presents the user with a drop-down list of miRNAs to choose from, resulting in a list of circRNA and its coordinates on the human genome, which might act as a sponge. Every circRNA has a corresponding UCSC genome browser link attached to it that displays the chromosomal location of the circRNA(**Figure 6A**). Also, an MGI page of RAG1 with 158 mouse RAG1 MGI hyperlinks to the expression of RAG1 in various tissues. The table consists of the RAG1 MG1 ID, the genes symbol “Rag1”, the link to the corresponding MGI database, the Assay type that determined the expression, the development stage of the mice at which the data was extracted, the tissue followed by the image id of the tissue expression data (**Figure 6B**).

### Interactive Interphases for the User

The following two utilities are highly user-friendly. The first is a Download section where all the data displayed on the database can be downloaded by the user with three available options, mouse data, human data, or all the files together (**Figure 6C**). One of the most exciting utilities comes next: the page where users can upload their own data and visualize it by adhering to the following steps. 1) It starts by asking you to upload a file of your choice in a tabular format by clicking on the “Browse button”, 2) If your input file has a header, you check the “Header” box followed by 3) choosing the separator that is present in your file (Comma, Semicolon or Tab) (**Figure 6D**). Once all these three steps are followed, the values of your file will be displayed in a tabular format followed by a heatmap depicting the same.

### Advantages of the Database

Using the database, miRNAs against RAG regulators can be identified, and their significance in regulating genomic integrity be studied. As an example, we would like to draw focus on miR-29c-3p. When gathering and analyzing expression data of miRNAs known to regulate RAG1 across B cell developmental stages for the database, we came across miR-29c-3p. We observed that the expression of this miRNA is negligible in the preB and proB cell stages, while it increases drastically in the mature stages of B cells, indicating an inverse pattern of expression. These findings were validated experimentally using knockdown and overexpression studies. It was also seen that in 87% of CLL patients and 79% of T-ALL patients, the expression levels of miR-29c-3p and RAG1 were inversely correlated (28).

## Discussion

RAG complex is a well characterized and well-studied nuclease complex protein that is involved in DNA damage and Repair mechanisms in more ways than one (43, 44). Specifically expressed in the early stages of B and T cell development, RAGs primarily initiate the process of V(D)J recombination (1, 3, 4, 9). Aberrant RAG expression, *via* various non physiological mechanisms, leads to translocations that in turn lead to leukemias and lymphomas (9, 14–16). Aberrant RAG can be blocked using certain means, such as microRNAs and regulatory transcription factors (19, 28). It is only imperative that these expression profiles be collated and studied to derive therapeutic gains from them. Here we present a database specific to RAGs that provides regulatory information in an NGS data analysis context. Apart from this database, Immgen hosts a broad-spectrum resource for gene expression and miRNA expression during normal haemopoietic development stages and cell types (45). miRAGDB database is focussed towards understanding crucial player in the immune system (RAG1 and RAG2) which accounts for the diversity in both cell mediated and humoral immunity by activating V-(D)-J recombination.

Gathering such information into one single resource can prove to be of much relevance. One example is the recently published study that highlights the role of miR-29c-3p in RAG1 expression in a stage specific manner and in CLL and T-ALL patients (28). miRAGDB further explores the factors involved in regulating RAGs.

The database contains information from Human and Mouse, normal B and T cell developmental stages. The sequencing data available from mice is much more than that in humans. From mice, miRNA expression data was available for both B and T cell developmental stages, whereas only the B cell stages have been found for humans. miRAGDB offers data visualization in a heatmap form and the form of individual bar graphs. The Human page allows users to add and remove miRNA based on which miRNAs’ expression profiles they might want to study. Not only miRNA, but as discussed earlier, transcription factors and other proteins are responsible for a gene’s regulation; therefore, we have incorporated a page that lets the users understand the expression profiles of RAG regulators in the conditions mentioned earlier. It is always important to be able to understand the expression profile and subsequently the possible function of a gene in the aberrant conditions; once the same is known in a normal scenario and hence the database also hosts expression profiles of transcription factors associated to RAG1 and miRNA known to regulate it in CLL and T-ALL patients.

Another valuable and unique featurette is the page where users are allowed to upload their own expression matrices and visualize data. This database is only the first version. We will incorporate additional information in the upcoming versions as we gather more information from various B and T cell sequencing experiments and other leukemia and lymphoma conditions. Meanwhile, this utility where one’s data can be uploaded can be used extensively by the users to view expression profiles from any experiment as they please, in the form of a comprehensive heatmap. Lastly, the database has links to MGI and UCSC genome browsers where information on alternate RAG1 and RAG2 transcripts in mice, mouse RAG1 and RAG2 phenotypes, associated circular RNA, etc. is available.

## Data Availability Statement

The content of the database and the software for generation of custom expression visualization are freely available at the URL http://52.4.112.252/shiny/miragdb/. Scientists interested in adding or curating data (miRNA/ regulator expression profiles) or in implementing options that are not yet available are encouraged to contact BC (at vibha@ibab.ac.in). This article should be cited in research projects assisted by the use of **miRAGDB**. The datasets presented in this study can be found in online repositories. The names of the repository/repositories and accession number(s) can be found in the article/**Supplementary Material**.

## Author Contributions

BC, SD, and SR conceived and coordinated the study. BC and SD obtained and analyzed the sequencing data. SW wrote the code to implement the database. BC, SD, and SW interpreted the data and wrote the manuscript. All authors contributed to the article and approved the submitted version.

## Funding

We thank the Department of Information Technology, Biotechnology and Science and Technology, Govt. of Karnataka, India, and Department of Science and Technology (SR/FST/LSI-536/2012), India for infrastructure grant and the Bio-IT grant, which helped in the design of the study, collection, analysis and interpretation of data and in writing the manuscript. SD is supported by Department of Biotechnology (Ref. no BT/PR13458/COE/34/33/2015 and BT/PR13616/GET/119/9/2015), Govt. of India, India.

## Conflict of Interest

The authors declare that the research was conducted in the absence of any commercial or financial relationships that could be construed as a potential conflict of interest.

## Publisher’s Note

All claims expressed in this article are solely those of the authors and do not necessarily represent those of their affiliated organizations, or those of the publisher, the editors and the reviewers. Any product that may be evaluated in this article, or claim that may be made by its manufacturer, is not guaranteed or endorsed by the publisher.

## Supplementary Material

Supplementary Figure 1 **(A)** Flowchart for methodology followed to extract and analyze the sequencing data **(B)** Depicts the working of Shiny R application in terms of reading the data files and visualizing it on the interface and **(C)** is a list of tools and R packages used to create the database.
