# Supplementary material for: MiRAGDB: A Knowledgebase of RAG Regulators
Source: Front Immunol. 2022 Mar 24;13:863110. doi: 10.3389/fimmu.2022.863110 (PMC8987502; doi:10.3389/fimmu.2022.863110)
Supplement: Supplementary Figure 1 — (A) Flowchart for methodology followed to extract and analyze the sequencing data (B) Depicts the working of Shiny R application in terms of reading the data files and visualizing it on the interface and (C) is a list of tools and R packages used to create the database. [file Presentation_1.pptx]

## Slide 1
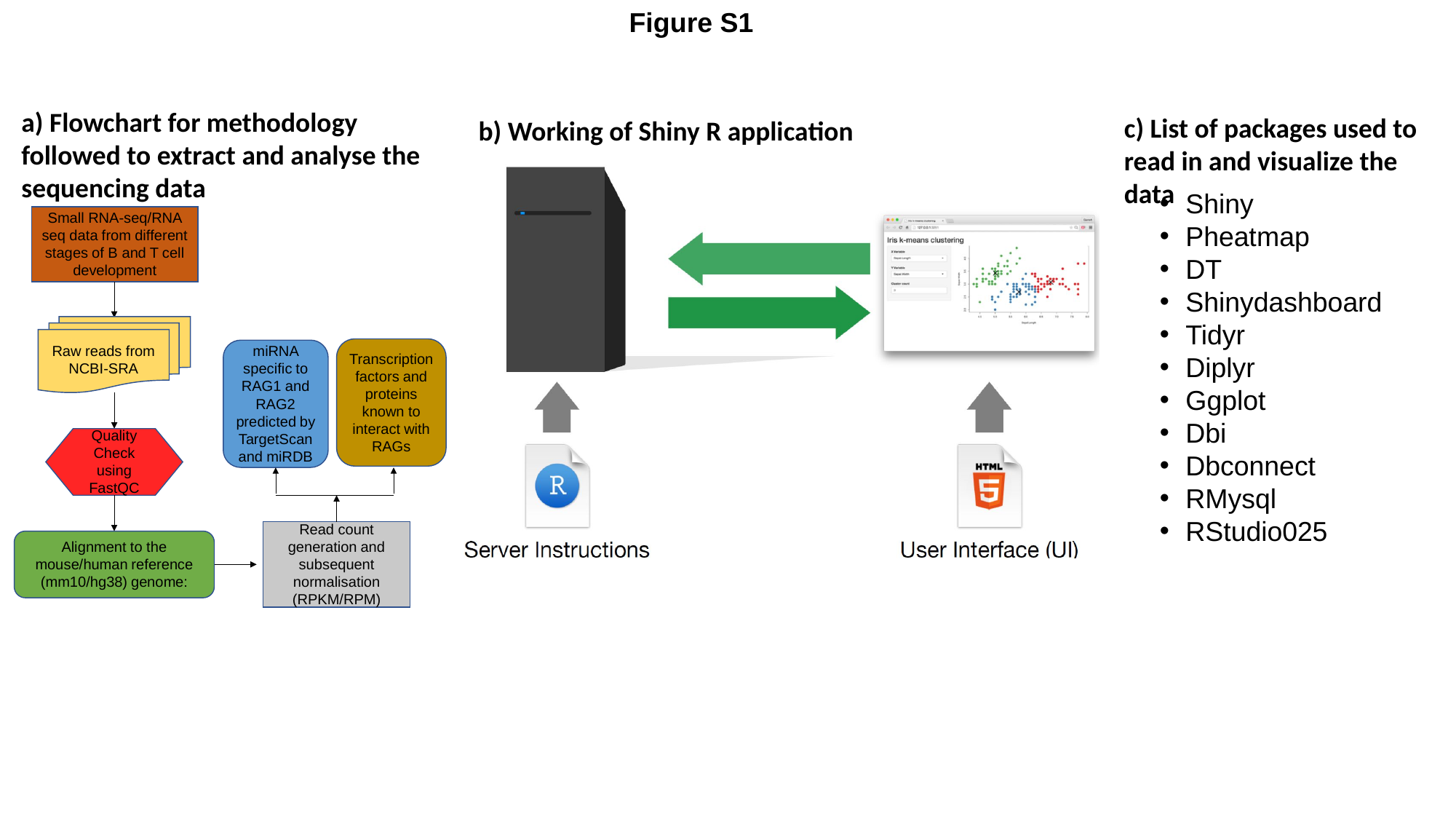

Figure S1
a) Flowchart for methodology followed to extract and analyse the sequencing data
c) List of packages used to read in and visualize the data
b) Working of Shiny R application
Shiny
Pheatmap
DT
Shinydashboard
Tidyr
Diplyr
Ggplot
Dbi
Dbconnect
RMysql
RStudio025
